# Reviewing the role of positive classroom climate in improving English as a foreign language students’ social interactions in the online classroom

**DOI:** 10.3389/fpsyg.2022.1012524

**Published:** 2022-10-21

**Authors:** Fei Qiu

**Affiliations:** School of Foreign Studies, Changzhou University, Changzhou, China

**Keywords:** online education, online L2 learning, positive classroom climate, social interactions, emotional health, communicative skills, affective well-being

## Abstract

The teacher and learners are cooperatively involved in the creation of a positive climate in an L2 class. In the online language learning environment today, teachers can make the best use of technology, multimedia learning, and accessibility of learners to create a supportive and effective climate. In this productive climate, the teacher and learners can have multiple forms of social interaction which can improve language learners’ communicative skills. Not only can the teacher expect better learning outcomes, but s/he can also ensure students’ wholehearted attendance in, attention to, and participation in class activities. A positive class climate and the consequent better social interactions can also enhance learners’ affective well-being. For example, higher self-esteem leads to lower levels of anxiety and better emotional health. This study aims to review the contribution of a positive classroom climate to the improvement of students’ social interaction in the online L2 classroom. To do this, the findings of the relevant studies have been presented and their implications for the construction of a positive online L2 classroom climate have been provided. Suggestions are made on how to help teachers create a positive climate in online L2 classes and how to pave the way for more effective social interactions between teachers and students and among students. Also, implications are provided for L2 teachers, researchers, and trainers, especially in the post-pandemic era.

## Introduction

With the advent of the positive psychology movement in the field of second language acquisition (SLA), the attention of scholars in this field has been drawn to the role of positive emotions, character strengths, and positive institutions in the microsystem of class interactions. Thus, the focus on the positive classroom climate seems to be a salient factor in this positive psychology movement. On the other hand, due to the pandemic issues, the online classroom environment has turned into the mainstream education system of language learning in the past few years. Compared to in-person classes, in this online environment foreign language learners might experience a gamut of feelings from anxiety to enjoyment and boredom (see Derakhshan et al., 2021; Pawlak et al., 2021; Yazdanmehr et al., 2021; Resnik et al., 2022; Dewaele et al., 2022a). Therefore, the incorporation of a positive classroom climate with the online environment of L2 classes seems to set the positive affective stage for the enhancement of online classroom interactions. However, one specific gap concerning the literature on the positive psychology of online L2 classes might be still the need for the delineation and clarification of the main strategies and pedagogical implications in this domain. Thus, a conceptual review of the relevant studies in the literature on positive classroom climate in language learning can pave the way for the implementation of the main strategies in this domain in the online L2 learning environment. Given this gap, the present paper shows interest in a systemic review of the contribution of a positive classroom climate to the constructive and effective interactions which can be incorporated into online L2 learning. To do this, it begins with an overall view of the classroom climate concept and elaborates on the relationship between classroom climate and fostering of social interactions. Then, it moves on to discuss the climate of online L2 courses and how it can be oriented toward a positive climate. More specifically, a review of the literature on L2 class climate and the associated social interactions is presented, which is followed by implications for online L2 classes. More specifically, several conclusive and summative remarks will be made on the promises of a positive classroom climate for fostering social interactions in online language learning. The paper will end with a number of suggestions for prospective researchers to fill the existing gap to further investigate the online L2 classroom climate and how it can change, as positively as possible, to host supportive social interactions and fruitful learning outcomes.

### Overview of classroom climate

The classroom climate reflects students’ assessment of their educational experience (Reid and Radhakrishnan, 2003; Khalfaoui et al., 2021; Derakhshan et al., 2022c). This assessment can include students’ opinions about the rigor of their class, the interactive opportunities with the teacher and other students in class, and engagement in class tasks and activities. Every student has a personal perception of the environment of the classroom. Yet, a group-based and collective perception and feeling can also be shared by the students and their teacher. Thus, the classroom climate can be seen as a shared sense of the environment of the class by the students attending the class (Alonso-Tapia and Ruiz-Díaz, 2022). The classroom climate is mainly comprised of student perceptions that grow under the influence of exposure to several learning contexts and the existing opportunities and resources in the environment which help to develop impressions and to make judgments accordingly (Fraser and Treagust, 1986). Classroom climate denotes the global atmosphere of a classroom which is constructed *via* different types of interactions in the classroom. These interactions involve both student–student and teacher–student interactions (Gazelle, 2006). The term “classroom climate” is associated with positive psychology as it has been depicted as a “positive climate” in which enjoyment, connectedness, and enthusiasm are incorporated in the classroom interactions (Reyes et al., 2012). The emergence of this positive climate is the result of teachers’ warm and supportive behavior (Hamre et al., 2013) based on which language development can be fostered (Cameron et al., 2008) and engagement in academic tasks can be enhanced (Klem and Connell, 2004). That is, a positive classroom climate is claimed to follow from both the teacher’s and students’ active engagement in social interactions, classroom rapport, and the co-construction of the learning atmosphere (Sidelinger and Booth-Butterfeld, 2010; Frisby et al., 2014; Derakhshan, 2021; Derakhshan et al., 2022b). There is research evidence that a lasting positive classroom climate and the teacher-student emotional bond can improve learners’ satisfaction, motivation, class participation, engagement in group tasks and activities, overall well-being, and reduce their anxiety and internal sense of fear (Graham and Gisi, 2000; Ellis, 2004; Hamre and Pianta, 2007; Norton, 2008; Barr, 2016). The necessity of such a positive climate lies in the fact that when language teachers and students interact in a positive atmosphere, they can have better concentration, feel calmer, achieve better educational goals, and work to their best (Elahi Shirvan and Taherian, 2020). Of note is that a major feature of a positive classroom climate is the enhancement of effective interpersonal relations (Bayat et al., 2020; Qin, 2022).

Barr (2016) draws attention to interpersonal interactions in class and how they can develop within a constructive and supportive classroom climate (Xie and Derakhshan, 2021). In a positive classroom climate, teachers develop positive interactions with their learners (Barr, 2016; Elahi Shirvan and Taherian, 2022). They also play a major role in directing constructive, supportive, and positive interactions as well as modeling support in class (Johnson, 2009; De Ruiter et al., 2019). Besides the pivotal role of teacher-learner interactions in the classroom climate, learner-learner interactions are salient in a positive classroom climate (Frisby and Martin, 2010; MacLeod et al., 2018). A socially active and productive classroom climate is marked by students interacting in supportive and sympathetic interactions with peers (Dwyer et al., 2004; Taherian et al., 2021). Student–student interactions are constructed based on certain behaviors such as appreciation, smiling, and sharing of thoughts and memories, which can positively affect learning experiences and achievements (Sidelinger et al., 2012; Elahi Shirvan and Talezadeh, 2018a; Vranjes and Brone, 2021; Lim et al., 2022). To further emphasize the flourishment of social interactions in a positive classroom climate, Hirschy and Wilson (2002) pinpointed that teaching and learning do not happen just between students and their teacher, but it occurs among students in class as well.

### Classroom climate and social interactions

A positive classroom climate for the best forms of learning to occur is marked by teachers trying to establish effective positive interactions in the environment of the class (Shapiro, 1993). As described by Shmuck and Schmuck (1975), in a positive classroom climate students expect each other to try their best intellectually and to support each other in doing class activities. The kind of effective social interactions is in the form of students sharing much influence with each other as well as with the teacher, and there are high levels of interest in group work as a whole and also among classmates (Shapiro, 1993). In such a positive climate, open communication is supported mainly in the form of dialogs and the existing norms support the academic work being done (Shapiro, 1993). However, it is noteworthy that teacher’s expertise cannot on its own, within a positive classroom climate, warrant effective social interactions. As maintained by Giles (1959), these constructive social interactions will not emerge within a positive classroom climate unless the goals, expectations, and values are clearly stated. When teachers’ ambiguities are lowered by recognizing every student’s talent, abilities, and expectations, teachers are better capable of managing socially effective interactions in class (Schmuck, 1968).

The benefit of the social interactions promoted in a positive classroom climate is that both the students and the teacher achieve a better understanding of each other and themselves. This enhanced understanding of each other’s values fosters a better establishment of the expectation culture and provides better chances for both teachers and students to move toward better academic achievements (Moos, 1973). Also, the social interactions developed in a positive class climate help to increase learners’ self-esteem and improve their chances for success (Anderson, 1970). This was how the pioneers in investigating positive classroom climate and its effects on class procedures perceived the topic. A similar trend exists in the L2 educational context, as will be reviewed below.

## Literature on positive L2 class climate and social interaction

Raghul and Saradha (2016) discussed that learning English is facilitated if the language learner’s interest is stimulated in class. Despite the presence of many stimulating sources for a language learner, the positive classroom climate itself is capable of motivating those students at a basic level of language proficiency to get actively involved in the process of language learning in class. A supportive classroom climate gives a plenty of energy to students to collaboratively learn the language; it also poses a positive effect on them and allows them to feel adequately comfortable in class to share their thoughts and intentions of learning. A positive L2 class environment increases one’s willingness to participate in classroom interactions, helps to develop a positive approach to learning, and can, thus, improve language learning outcomes. Raghul and Saradha (2016) continued to describe a friendly classroom climate as attractive to L2 learners and helpful for them to develop their confidence to get socially active in class. Such a climate strengthens L2 learners’ enthusiasm for language learning and can even turn L2 learners’ negative attitudes toward classroom-based language learning. A positive L2 classroom climate also increases language learners’ self-esteem, which can significantly affect their participation in classroom interactions. It is worth noting that language learners’ achievement can be significantly influenced by their teachers’ feedback through different strategies, such as using innovative teaching aids (including online sources), with a focus on teacher–student interactions, and providing individually tailored guidance in a more effective learning environment (Raghul and Saradha, 2016).

Gabryś-Barker (2016) drew attention to how positive psychology, which is based on humanistic assumptions, can be maximally used by L2 teachers to increase L2 learners’ active role in class, their learning outcomes, and also their affective development (Wang et al., 2021). One of these contributing factors is the positive climate of the L2 class. Gabryś-Barker (2016) investigated the role which the L2 class climate plays in language teacher’s and learners’ achievements, personal growth, interactions, and well-being. These researchers began with an introduction to the classroom climate concept and reviewed exemplary works of research on positive class climate. In their study which was carried out among pre-service English as a foreign language (EFL) teachers, they explored the teachers’ awareness of the features of positive classroom climate, their perception of teacher’s and learners’ role in constructing this climate and the preservice teachers’ perceived importance of classroom climate for the teacher–student interactions. The findings pointed to different trainees’ perceptions of the constituent elements of a positive class climate and the effect of positive class climate on teacher–student rapport, learning outcomes, and well-being.

One way to create a positive class climate in L2 learning and enhance teacher–student social interactions and rapport in class is through teacher self-disclosure (Elahi Shirvan and Taherian, 2020; Qin, 2022). Teacher self-disclosure means a teacher’s verbal narration of personal accounts about his/her life that can or cannot be relevant to the main content of the syllabus but provides such personal information that is unlikely for students to acquire from others (Sorensen, 1989). Teacher self-disclosure has been revealed to have different positive effects on L2 classroom learning and creating a friendly and open atmosphere in class to increase language learners’ social interactions, engagement in discussions, enthusiasm for L2 learning, and class participation (Cayanus and Martin, 2004; Henry and Thorsen, 2021; Cui, 2022). Furthermore, teacher self-disclosure has been proven effective in paving the way for establishing a positive and supportive classroom climate to increase teacher-learner rapport and enhance students’ affective and emotional health (Grewe, 2017; Weber et al., 2021).

Another integral component of a positive classroom climate especially for language learning, as derived from the literature, is the immediacy between the teacher and students (Gedamu and Siyawik, 2015). This concept was initially introduced by Mehrabian (1969) as a communication trait to represent the extent to which individuals are close to each other in an environment (Finn and Schrodt, 2012). Immediacy entails different verbal and non-verbal manifestations and techniques that students and teachers often use in class to achieve a feeling of cohesiveness and closeness to each other (Dickinson, 2017; Delos Reyes and Torio, 2020; Derakhshan et al., 2022a). There is research evidence for the advantages of improving immediacy in L2 classes to induce empowerment for human actors (teacher and students; Cakir, 2015), social interactions (Marx et al., 2016), reduced ambiguity in communicative tasks (Zheng, 2021), focused attention (Bolkan et al., 2017), and lower anxiety (Kelly et al., 2015). More recently, Derakhshan et al. (2022c) investigated a model of classroom social climate, growth language mindset, boredom, and student engagement among Iranian EFL learners in an online setting. Their use of structural equation modeling (SEM) demonstrated that classroom social climate and boredom significantly predicated EFL students’ engagement directly. Nonetheless, the growth language mindset impacted student engagement through the mediating effect of boredom.

### Online classroom climate

Online education, playing a complementary role in conventional mainstream before the Covid-19 pandemic (see Shahnama et al., 2021a), has now become, more than ever before, dominant in global education systems (Tesar, 2020), including the language learning domain. This sudden extensive use of online learning could even be at the expense of quality if adequate pedagogical practices were lacking in the classroom environment (Sithole et al., 2019). There can be certain advantages and disadvantages to teaching online classes with their idiosyncratic contextual features. In a dominantly online class, all the materials (feedback, assessments, etc.) are delivered *via* a Learning Management System (LMS) with an environment specially designed for educational purposes (Franks, 2002; Hiltz and Turoff, 2005; Parsad and Lewis, 2008). Thus, there is a growing dependence on using different platforms with different environments for social interaction and social networking to facilitate student learning (Franks, 2002; Alstede and Beutell, 2004). It is noteworthy that this trend is driven by ever-increasing demands for flexibility (on the part of teachers and learners both) and the need for an adaptive and supportive classroom climate. Still, concerning the requirements of online education and more specifically language classes, teachers’ and learners’ challenges in developing a constructive classroom climate remain under-researched (Sithole et al., 2019).

The online education promoted by the pandemic has put much pressure on students and teachers both, and has somehow threatened their cognitive and affective well-being (Casañ-Núñez, 2021; Shahnama et al., 2021b; Dewaele et al., 2022a). Regarding the construction of a positive climate of foreign or second language learning, and given the interplay of cognition and emotion, underlying the positive psychology movement of SLA, teachers should be encouraged to employ different strategies to create a positive climate in online classes to develop positive emotions and effective interactions in which learning is mixed with pleasure (Casañ-Núñez, 2021). This means that there is a need for more elaborations on how the online L2 class climate can be turned into a positive climate in terms of the interactions happening between and among the main human actors involved (i.e., the teacher and students). To do this, the main themes of the studies on positive L2 class climate and social interactions will be considered as pedagogical implications and suggestions for the construction of a positive climate in online L2 classes.

### Implications of positive classroom climate for online L2 classes

The pre-pandemic focus of individual differences hosted a context-independent or decontextualized body of research, which has been gradually substituted by an interaction view that takes into account the environment or the climate where individual differences manifest themselves in student–student and teacher–student interactions (Dewaele and Thirtle, 2009; Dörnyei and Ryan, 2015; Gkonou, 2017; Williams et al., 2019). Thus, given the ecology of a positive classroom climate in mind, it is necessary for language teachers to explore the complex interactions among language learners, how they make use of the supportive elements of their class climate, and the atmosphere in which they acquire the language, either physical or virtual (Ng, 2021). In recent years, marked by a rapidly growing use of online L2 learning, the prevalent use of mobile and portable devices as well as the use of wireless technology for language learning have instigated more interest in using these technologies in online language learning programs too (Kohnke and Moorhouse, 2022). Due to the unique setting of online language classes, language learners might experience different positive and negative emotions in this setting (Dewaele et al., 2022a). Therefore, given the recent positive psychology movement in the field of SLA, turning this setting into a positive climate can take place *via* a reflection and adoption of the main techniques and strategies introduced in the literature of positive climate and social interactions.

Apart from the mobility feature, a positive classroom climate featuring these technologies is marked by the possibility of incorporating multiple media (different sources of audio-visual and textual information), which facilitates more effective teacher-learner and learner-learner interactions (Traxler, 2009; Manegre and Sabiri, 2022; Moorhouse et al., 2022). The multimedia-laden L2 classroom climate has led to the development of an interactive course content and cooperative learning tasks to complete in several settings. For instance, L2 learners can use various applications and social media on their mobile device and share ideas and thoughts with other online learners through formal or informal chatting, and textual or audio-visual chats in class, which has made them more socially active than before (Hsu and Ching, 2012; Barrot, 2021). In other words, the technology-laden climate of online L2 classes can use all the resources, especially the multimedia facilities, to their best to foster interpersonal interactions (Lai and Tai, 2021). Much of the language learning outcome and communication skills can emerge out of these technology-facilitated environments (Xue and Churchill, 2022).

Choi and Chung (2021) investigated how teachers newly experiencing the online L2 classes tried to create a positive classroom climate to foster effective interactions among students. Two strategies commonly used by the teachers were (a) increasing the sense of social connectedness by motivating language learners to be present in the virtual class environment and be as accessible as possible, and (b) assigning goal-oriented and technology-mediated tasks and activities with long-lasting effects. Other researchers have also pinpointed that social presence is essential to increase online students’ level of cooperation and satisfaction with the online learning experience (e.g., Gunawardena and Zittle, 1997; Richardson and Swan, 2003; Hostetter and Busch, 2006; Arrosagaray et al., 2019; Slim and Hafedh, 2019; Esra and Sevilen, 2021). The element of social presence means the student’s perceived gist of the online group and the predominance of intimacy and proximity experienced with peers or the teacher in a technologically rich communication context (Richardson et al., 2017). As the definition shows, this social presence seems to be an essential feature of an effective L2 class climate and, if managed well by the teacher, it can be translated into effective interpersonal communication in class.

More specifically, three suggestions were made by Choi and Chung (2021) to help create an effectively sustainable online L2 classroom climate. One was to adequately train language teachers to efficiently use interactive online LMSs, applications, and Web 2.0 tools to ensure language learners’ active participation in collaborative interactions in class. The second was to foster the collective responsibility concept among language teachers to motivate them to take part in teachers’ sustained professional interactive events. The third one was to welcome self-initiated communities of practice in which teachers could cooperatively consult with others and take part in continuous professional development. These suggestions could help EFL teachers in online courses to act more effectively and professionally in class to facilitate productive interactions in an amiable and supportive climate.

## Conclusion

A classroom climate has been viewed as a collective and subjective perception of the class, its features, actors (those involved), and their social interactions (Weber et al., 2021). In other words, classroom climate pertains to the positive or negative teacher’s and learners’ predominant moods and attitudes toward a classroom. A positive classroom climate is supportive and welcoming, whereas a negative classroom climate is harmful and destructive for teachers and their learners. As derived from the literature, there are different ways that a positive L2 classroom climate can promote social interactions in online L2 classes. One is by creating an environment in which students feel comfortable enough to share their thoughts with peers and their teacher. A positive online classroom climate can increase language learners’ willingness to participate in cooperative tasks and group works. As already pinpointed, a positive online L2 classroom climate helps to develop the self-confidence needed to express one’s thoughts freely in class. In this way, the L2 teacher’s feedback plays a key role, especially if this feedback is given through innovative teaching aids and facilities, such as the multimedia present in online classes. Also, given the literature, a positive classroom climate can be constructed *via* teacher’s self-disclosure, which helps to establish a warm and welcoming climate for all students to actively engage in online social interactions.

Another feature of a positive L2 classroom climate that could lead to better online social interactions has been found to be the immediacy between teachers and their learners, which pulls them close enough to share thoughts and ideas clearly and willingly. In online L2 classes, teachers are recommended to make the best use of multimedia resources to attract their students’ attention to and interest in the content and cooperative tasks. They are also recommended to maximize language learners’ social presence and to emphasize their accessibility in the social atmosphere of the online class. Moreover, they are supposed to assign goal-oriented and technology-mediated cooperative tasks and activities to pull all seemingly departed online learners of the class together and create a cooperative and supportive climate to foster student engagement. Finally, online L2 teachers, especially novice teachers, are recommended to take part in training courses to develop sustained interactive events to learn how to act effectively to facilitate productive social interactions in an amicable and supportive climate in online classes (Teo et al., 2022).

What has been commonly found in the existing literature is that the creation of a positive online L2 class climate should be a shared responsibility between the teacher and learners. Evidently, the teacher’s role in guiding students to develop a positive attitude toward their language learning experience is of a paramount importance for the construction of this positive climate. Especially in an online L2 class, where students’ physical presence in the same learning environment is lacking, the teacher’s role becomes more important in keeping everybody actively engaged in classroom events. If all the available up-to-date resources are used appropriately, the social presence of students is emphasized, the teacher-learner immediacy is warranted, the teacher is self-disclosing enough, the appropriate feedback is provided through innovative multimedia channels, and a positive climate can be expected to follow, out of which online effective social interactions emerge.

### Directions for further research

With respect to the construction of a positive online L2 climate, it should be noted that different platforms are used for delivering online L2 course content. Also, different mobile applications, social media, and LMSs are increasingly used for this purpose. Each is featuring certain optional facilities for the online or offline sharing of the educational content. The nature of interactions facilitated by each application or platform may differ from the others, which needs to be cross-compared in future studies. Especially, the effectiveness of the different virtual environment each L2 online platform creates, in ensuring a positive classroom climate, is worth investigating in cross-comparative designs or case studies.

As already reviewed, some studies drew attention to the effect of a positive classroom climate on increasing such positive emotional or personality traits as self-confidence, grit, and emotional well-being and reducing negative emotions such as anxiety. These variables need to be explored in the online L2 classes in the post-pandemic era considering the current dearth of research with this respect. Still, many psychological variables are left unexplored in the potentially positive classroom climate of an online L2 class. Examples are mindset, foreign language enjoyment (FLE), foreign language learning boredom (FLLB), and compassion. Moreover, investigating the co-development of each of these variables in both the teacher and learners in the potentially positive climate of online L2 courses is left unexplored. To investigate this, there is a need for sophisticated qualitative and quantitative research methods or a mixture of both, which adequately meet the needs of a complex dynamic system of language learning. Examples of these innovative research approaches to guide the future line of related research are longitudinal growth curve modeling and its varieties (to exemplify quantitative methods) and process-tracing and ecological momentary assessment (as instances of qualitative research methods).

As suggested in the previous sections, L2 teachers need training on how to establish a positive classroom climate in online L2 classes. It is suggested that these training be developed in the presence of both novice and expert language teachers to share their experiences. Also, the quality and effectiveness of these training courses need to be investigated. Furthermore, the strategic content of these training courses for online L2 classes needs to be engineered by the field specialists and practitioners. The syllabus of these training courses can include (a) how to maximize learners’ social presence, (b) how to ensure immediacy among the teacher and students, (c) how to enhance teacher–student rapport, (d) how to increase teacher self-disclosure, (e) how to maximally use multimedia sources, and (f) how to maximally use technology-mediated cooperative tasks and activities.

1. Probably, there is a need for devising teacher mentorship as well to ensure that novice L2 teachers adequately learn how to develop a supportive and welcoming classroom climate for students to warrant a warm and willing participation in online social interactions in an L2 class. In addition, the structural plan of these mentorships needs to be engineered by L2 researchers and practitioners and their effectiveness should be evaluated. Furthermore, we need more ecological studies in the future to explore the potential barriers to the establishment of a positive classroom climate in online L2 courses. There is a wide gap with this concern in the L2 literature in the post-pandemic era. A sound knowledge of these potential inhibitive factors can guide L2 practitioners to find ways to reduce or remove barriers and; thus, pave the way for creating an effective classroom context that hosts effective social interactions and probably better learning outcomes.

## Author contributions

The author confirms being the sole contributor of this work and has approved it for publication.

## Conflict of interest

The author declares that the research was conducted in the absence of any commercial or financial relationships that could be construed as a potential conflict of interest.

## Publisher’s note

All claims expressed in this article are solely those of the authors and do not necessarily represent those of their affiliated organizations, or those of the publisher, the editors and the reviewers. Any product that may be evaluated in this article, or claim that may be made by its manufacturer, is not guaranteed or endorsed by the publisher.
